# The comparison of functional status and health-related parameters in ovarian cancer survivors with healthy controls

**DOI:** 10.1007/s00520-024-08311-x

**Published:** 2024-01-22

**Authors:** Sukriye Cansu Gultekin, Ahmet Burak Cakir, Zeynep Gulsum Guc, Faruk Recep Ozalp, Merve Keskinkilic, Tugba Yavuzsen, Husnu Tore Yavuzsen, Didem Karadibak

**Affiliations:** 1 Faculty of Physical Therapy and Rehabilitation, Graduate School of Health Sciences, Dokuz Eylul University, Izmir, Turkey; 2 Department of Medical Oncology, Izmir Katip Celebi University, Izmir, Turkey; 3 Department of Cardiopulmonary Physiotherapy-Rehabilitation, Faculty of Physical Therapy and Rehabilitation, Dokuz Eylul University, Izmir, Turkey; 4 Clinic of Gynecology and Obstetrics, Buca Obstetrics Gynecology and Pediatrics Disease Hospital, Izmir, Turkey

**Keywords:** Ovarian cancer, Functionality, Respiratory muscle strength, Health parameters

## Abstract

**Purpose:**

The primary purpose of this study was to evaluate functional status and health-related parameters in ovarian cancer (OC) survivors and to compare these parameters with healthy controls. The secondary purpose of this study was to compare these parameters in early and advanced OC survivors.

**Methods:**

Thirty-two OC survivors (*n* = 15 early stage; *n* = 17 advanced stage) with no evidence/suspicion of cancer recurrence after completing adjuvant local and systemic treatments for at least 12 months and 32 healthy controls were recruited for functional- and health-related assessments. Participants were assessed using the following methods of measuring the following: 6-min walk test (6MWT) for functional exercise capacity, 30-s chair stand test (30 s-CST) for functional fitness and muscle endurance, a handheld dynamometer for peripheral muscle strength, and a handheld dynamometer for lower extremity strength, Medical Micro RPM for respiratory muscle strength, International Physical Activity Questionnaire-Short Form (IPAQ-SF) for physical activity level, and Eastern Cooperative Oncology Group Performance Scale (ECOG-PS) for performance status, Checklist Individual Strength (CIS) for fatigue, Treatment/Gynecological Oncology-Neurotoxicity (FACT/GOG-NTX) for neuropathy, the Hospital Anxiety and Depression Scale (HADS) for anxiety and depression level, and the World Health Organization-Five Well-Being Index (WHO-5) for generic quality of life.

**Results:**

All OC survivors underwent surgery and chemotherapy, and only 9.4% received radiotherapy in addition to chemotherapy. The median recurrence-free period post-completion of adjuvant treatments was 24.00 (12.00–75.00) months. OC survivors had lower 6MWT (m) (*p* < 0.001, *r* = 1.50), peripheral muscle strength (*p* = 0.005, *r* = 0.72), knee extension (*p* < 0.001, *r* = 1.54), and respiratory muscle strength (maximal inspiratory pressure) (*p* < 0.001, *r* = 1.90) (maximal expiratory pressure) (*p* < 0.001, *r* = 1.68) compared to healthy controls. HADS-A (*p* = 0.005, *r* = 0.75) and CIS scores (*p* = 0.025, *r* = 0.59) were also higher in the OC survivors. Early-stage OC survivors had better 6MWT (m) than advanced-stage OC survivors (*p* = 0.005, *r* = 1.83). Peripheral muscle strength was lower in advanced-stage OC survivors (*p* = 0.013, *r* = 0.92). FACT/GOG-NTX scores were higher in early-stage OC survivors (*p* < 0.001, *r* = 1.42). No significant differences were observed between early- and advanced-stage OC survivors in other measures (*p* < 0.05).

**Conclusion:**

The findings suggest functional status, and health-related parameters are negatively affected in OC survivors. Additionally, higher levels of fatigue, neuropathy anxiety, and depression were reported in advanced OC survivors.

## Introduction

Ovarian cancer (OC) is one of the most common gynecologic malignancies, with approximately 240,000 new cases reported annually worldwide, and accounts for the highest mortality rate among gynecologic cancers [1]. The incidence of women diagnosed with OC is expected to increase by approximately 37% by the year 2040 according to GLOBOCAN cancer data [1]. Almost 60% of women with OC are not diagnosed until the disease has progressed to the advanced stage (III or IV), significantly increasing the risk of recurrence and premature mortality [2]. The introduction of definitive screening modalities and improved genetic and epidemiological tests to predict OC risk have contributed to the early detection of the disease in recent years [3]. The development of various treatment strategies also improved survival rates in OC during the last 20 years [3]. Thus, the importance of evaluating short- and long-term consequences of disease-related conditions and treatment side effects increases as both a clinical and a research endpoint with increased early detection and survival rates in OC [4, 5].

The primary treatment options for OC are local (debulking /cytoreduction surgery) and systemic (adjuvant and/or neoadjuvant chemotherapy) [2]. On the other hand, the comprehensive management of OC combines different implementations of varied approaches in accordance with the disease type, stage, and progression such as targeted drug therapy, hormone therapy, and radiotherapy in addition to these primary options [2]. OC patients commonly experience a varied range of physical and psychological symptoms resulting from the chronic disease process and the adverse effects of these treatments [2, 6]. These symptoms and related consequences include neuropathy, loss of muscle strength, fatigue, decreased mobility, exercise intolerance, dyspnea, and diminished health-related quality of life [7, 8]. Furthermore, the adverse effects of local and systemic treatments on health-related components of physical fitness (muscle strength, exercise capacity, body composition) may still persist even during the survival phase of OC [8–10]. Additionally, cancer accelerates aging and induces cellular inflammatory processes, damaging major organ systems [11]. The negative effects of this debilitating disease on general health result in a vicious cycle limiting the functionality of the patients which increases the risk of physical impairment in the years following cancer treatment. OC survivors commonly experience multiple comorbidities and often report limited engagement in physical activity, indicating impaired physical function and mobility in this population [12]. However, current knowledge gaps regarding the health-related parameters and functional level of OC survivors challenge the development of tailored cancer rehabilitation programs [13].

The importance of monitoring the physical and psychological side effects of treatment in cancer survivors is also emphasized in recent studies [13–15]. Although activity behaviors and physical function were compared in a preliminary study conducted with advanced OC survivors and controls [16], no studies comprehensively evaluated the effects of treatment and/or outcomes of disease processes in early and advanced OC survivors. Therefore, the primary aim of this study was to compare functional status (functional exercise capacity, functional fitness, and muscle endurance) and health-related parameters (peripheral, lower extremity and respiratory muscle strength, physical activity level, fatigue, neuropathy, level of depression and anxiety, and quality of life) of early stage (stage I or II) and advanced stage (III or IV) OC survivors and healthy controls. The secondary aim was to compare these parameters between early-stage OC survivors and advanced-stage OC survivors.

## Materials and methods

### Study design

The present comparative study was conducted as a multicenter, cross-sectional study involving three institutions: (Dokuz Eylul University Hospital Department of Medical Oncology, İzmir Katip Celebi University Atatürk Training and Research Hospital Department of Medical Oncology, and Dokuz Eylul University Faculty of Physical Therapy and Rehabilitation). This study was conducted between September 2022 and June 2023, and informed consent was obtained from all participants. The ethical approval for the study was acquired from the Noninvasive Research Ethics Board of Dokuz Eylul University (decision no.: 2022/29–05, date: 14 September 2022) and was carried out according to the Declaration of Helsinki.

### Participants

A total of 65 patients were informed about the objectives and method of the study and were screened for eligibility criteria during routine follow-up appointments and asked to participate. The patient population consisted of 32 OC survivors depending on inclusion and exclusion criteria. The control group consisted of 32 healthy participants among the relatives/acquaintances of the patients and the researchers. Inclusion criteria for OC survivors were being female with histologically confirmed OC, 18 years of age or older, volunteering to participate, having no evidence/suspicion of cancer recurrence after completing adjuvant local and systemic treatments for at least 12 months, and being able to read and understand the Turkish language. The exclusion criteria for OC survivors were having any musculoskeletal, cardiovascular, neurological, or other medical condition that could impair the assessments determined by their medical practitioner and ECOG-PS score of 3 and above (3: mobilization with assistive device, 4: bed depending). The inclusion criteria for healthy subjects were as follows: volunteering to participate, being 18 years of age or older, and being able to read and understand the Turkish language. Exclusion criteria for healthy subjects were as follows. Exclusion criteria for healthy subjects were as follows: having any diagnosed mental or physical diseases requiring regular or frequent medication [17].

### Study sample

The sample size was determined using G*Power software (ver. 3.1.9.6, Düsseldorf University, Germany). A priori power analysis was performed using data from a previous study that compared the activity behaviors of women with advanced OC to healthy controls [16]. The effect size of the difference in activity behavior between the two groups was calculated as 0.72. The minimum sample size of the study was estimated as 32 OC survivors and 32 healthy controls with a two-tailed test, a 5% type 1 error rate, and a minimum power of 80%.

### Assessments

A comprehensive medical history was obtained from all participants, including demographic information (age, gender, height, weight, and BMI), comorbidities, exercise habits, family history of cancer, and disease-specific characteristics including OC stage, disease duration, type of systemic, local treatment and medication use, and current symptoms (exercise-induced dyspnea, fatigue).

### Outcome measures

#### Functional exercise capacity

The 6-min walk test (6MWT) has been considered a reliable and valid test to assess functional exercise capacity according to the American Thoracic Society (ATS) recommendation [18]. The 6MWT was performed in accordance with ATS guidelines. Every minute, a standardized encouragement was given to the participants such as the following: “You are doing well,” or “Keep up the good work.” The 6-min walking distance (m) was recorded at the end of the test, and participants were not allowed any devices that assist walking during the test. The physiological measurements including heart rate, peripheral oxygen saturation (measured with a Jumper pulse oximeter, Germany), and systolic and diastolic blood pressures (measured with an Erka Manual Sphygmomanometer, Bad Toelz, Germany) were recorded both before and after the 6MWT. Perceived dyspnea severity and quadriceps femoris fatigue during the 6MWT were evaluated using a numeric rating scale.

#### Functional fitness and muscle endurance

The 30-s chair stand test (30 s-CST) was used to assess functional fitness and muscular endurance. Participants started in a seated position with their arms crossed. Participants were required to perform as many sit-to-stand repetitions as possible within 30 s. The number of full sit-to-stand repetitions of the participants within 30 s without using their arms was recorded. 30 s-CST is a reliable and valid tool for assessing functional fitness and muscle endurance in cancer survivors [19].

### Muscle strength

Hand grip strength was measured with a Jamar hydraulic hand dynamometer, a widely recognized and used device. The measurements were conducted using the standard position recommended by the American Society of Hand Therapists (ASHT) [20]. Three consecutive measurements were performed with a 1-min rest period in between, and the highest result in kilograms was used in analyses.

The handheld dynamometer (Lafayette Model-01165 and Hoggan microFET2) is a validated assessment tool for evaluating muscle strength [21]. Based on previous research showing excellent reliability, standard test positions were used to measure maximal isometric strength during knee extension [21]. Participants were instructed to maintain their maximum isometric contraction for 5 s at the endpoint following the movement. The highest score from three consecutive measurements taken at 1-min intervals was recorded. Strength measurements were recorded for both extremities, and the maximum of the measurements was taken.

### Respiratory muscle strength

Respiratory muscle strength was measured using a portable device (Micro Medical Micro RPM, UK) according to ATS criteria [18]. Intraoral pressure measurement, a noninvasive valid and reliable method, was used to assess maximum inspiratory pressure (MIP) and maximal expiratory pressure (MEP) [18]. For MIP measurement, the participants were first asked to perform maximal expiration followed by maximal inspiration for 1–3 s with the airway closed by a valve. For MEP measurement, the participants were first asked to perform maximal inspiration followed by maximal expiration for 1–3 s. Both measurements were performed in the sitting position and with the nose clip on. At least five measurements were made, and the highest of the three best values was recorded, with no more than 10% difference between the three values. Predicted values for respiratory muscle strength were calculated using equations according to age and weight [18].

### Physical activity measurement

The level of physical activity in the previous 7 days was evaluated using the International Physical Activity Questionnaire-Short Form (IPAQ-SF). IPAQ-SF includes seven questions about time spent sitting, walking, doing moderate-intensity exercises, and vigorous activities. The score is calculated by multiplying the minutes, days, and MET values of these activities. Participants were also classified into different physical activity subgroups according to their IPAQ-SF outcomes. Physical inactivity is defined as having a physical activity level of less than 600 MET-min/week, low physical activity is defined as having a level between 600 and 3000 MET-min/week, and sufficient physical activity is defined as having a level over 3000 MET-min/week [22]. The Turkish version of IPAQ-SF has been demonstrated valid and reliable tool for evaluating the physical activity levels [22].

### Performance status

The ECOG-PS (Eastern Cooperative Oncology Group Performance Status) scale was used to assess the performance status of OC survivors. The Turkish version of ECOG PS is a reliable and valid tool for assessing the performance status of patients with gynecologic cancers [23]. The ECOG-PS classifies the performance status of patients on a scale ranging from 0 to 5, with each category representing a different level of functional ability and care requirements [23].

### Fatigue

The Checklist Individual Strength (CIS) was utilized for assessing fatigue levels. The CIS is a valid and reliable tool to evaluate fatigue in cancer patients. The questionnaire consists of 20 items and four dimensions: subjective feeling of fatigue (eight items), motivation (four items), physical activity (three items), and concentration (five items). A higher score on the CIS indicates a greater degree of fatigue, decreased motivation, reduced physical activity, and concentration difficulties [24]. The Turkish version of the CIS is a valid and reliable instrument for assessing fatigue [24].

### Neuropathy

Neuropathy Functional Evaluation of Cancer Treatment / Gynecological Oncology-Neurotoxicity (FACT/GOG-NTX) was used for the assessment of peripheral neuropathy symptoms, including sensory, motor, and auditory problems, and sensitivity to cold. It consists of 38 questions and 5 subscales evaluating physical status (7 items), social life and family status (7 items), emotional status (6 items), and activity status (7 items). Neuropathy-specific concerns are measured by the “other concerns” subscale (11 items) [25]. The FACT/GOG-NTX total score is obtained by summing the scores of these five subscales. The total scores range from 0 to 152, and a higher total score indicates more severe neuropathy and a higher impact of neuropathy on health-related quality of life. The FACT/GOG NTX is a reliable and valid tool for evaluating neuropathy in cancer patients [25].

### Anxiety and depression

The anxiety and depression level of participants was assessed using the Hospital Anxiety and Depression Scale (HADS). The Hospital Anxiety and Depression Scale (HADS) is divided into two subscales: HADS-A, which identifies anxious states, and HADS-D, which identifies depressive states [26]. Each subscale consists of seven items. Subscale scores range from 0 to 21, with higher values indicating more anxiety or depression [26]. Participants respond to each item based on their recent feelings and activities. The HADS has shown appropriate psychometric properties for assessing the emotional state of cancer patients [26].

### Quality of life

Generic health-related quality of life was evaluated using the World Health Organization-Five Well-Being Index (WHO-5). The WHO-5 has validity and reliability as a generic scale for the measurement of well-being in the previous 14 days [27]. WHO-5 is a 5-point Likert-type scale, and each item is scored between 0 “not at all” and 5 “all of the time.” The overall raw score ranges from 0 (absence of well-being) to 100 (the highest level of well-being). The Turkish version of the WHO-5 has been demonstrated to be a reliable and valid tool for assessing health-related quality of life [27].

### Statistical analysis

The statistical analysis was carried out using IBM SPSS Statistics for Windows (version 20.0, Armonk, NY, USA: IBM Corp.). The statistical significance level was set at *p* < 0.05. The normal distribution of the data was determined using the Shapiro–Wilk test, kurtosis-skewness statistics, detrended Q-Q plots, and histograms. Descriptive statistics were presented as means and standard deviations (SD) for continuous variables with normal distributions and as frequencies and percentages (n%) for categorical variables. The ordinal parameters were compared between OC survivors and healthy control groups and in subgroup analysis (comparing early and advanced OC) using an independent *t*-test or Mann–Whitney *U*-test. Categorical variables were compared between the groups using the chi-square test or Fisher’s exact test. Effect sizes (d) were calculated using means and standard deviations for normally distributed data and Z-scores for non-normally distributed data. Cohen’s guidelines were followed to interpret effect sizes as small (*d* = 0.10–0.29), moderate (*d* = 0.30–0.49), or large (*d* = 0.50–1.00) [28].

## Results

### Participant’s characteristics

**Fig. 1:**
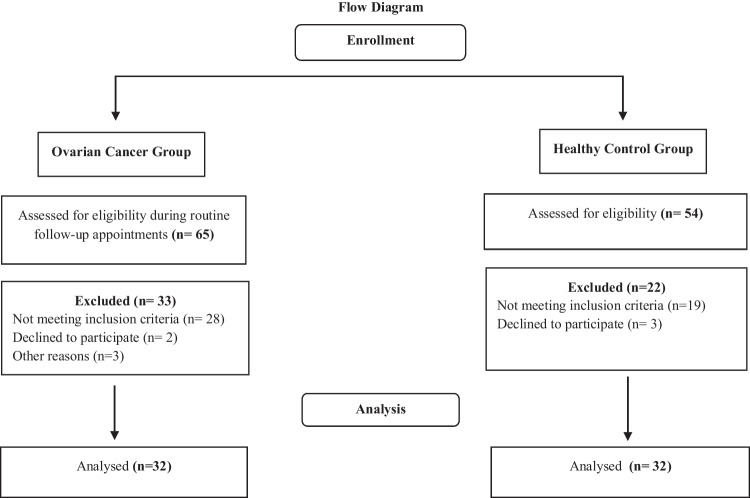
Study flow diagram

The inclusion criteria were met by 32 (49.23%) of the 65 screened OC patients. Thirty-three patients declined for various reasons: 15 declined to participate, 8 had an ECOG-PS score of 3 or higher, and 10 had a physical impairment limiting mobility in the lower limb. Among the 54 healthy adults assessed for eligibility, 19 individuals were excluded from the analysis to ensure comparability between groups. Consequently, the final study cohort consisted of 32 OC survivors and 32 healthy adults (Fig. 1). Thirty-two OC survivors (mean age: 54.43 ± 11.51 years; BMI: 28.56 ± 5.96 kg/m^2^) and 32 healthy controls (mean age: 50.93 ± 8.21 years; BMI: 27.26 ± 4.60 kg/m^2^) were included in the study. Comorbidities were present in 50% of patients with OC survivors. Among these patients, 71.5% had diabetes or arterial hypertension, and 28.5% had cardiovascular disease. A total of 46.8% of the OC survivors (*n* = 15) had early stage (stages 1 or 2), and 53.2% (*n* = 9) had advanced stage (stages 3 or 4) disease based on the International Federation of Gynecology and Obstetrics (FIGO) classification. All OC survivors in the study received surgical resection and chemotherapy. Only three patients (9.4%) had radiotherapy with adjuvant chemotherapy. The median time without evidence of cancer recurrence after completing adjuvant local and systemic treatment was 24.00 (12.00–75.00) months. The demographic, health-related, and disease-specific characteristics of the participants are shown in Table 1.

**Table 1:** Comparison of demographic and clinical characteristics of ovarian cancer survivors and healthy controls

	Ovarian group (*n* = 32) Mean ± SD Median (min–max) *n* (%)	Healthy group (*n* = 32) Mean ± SD Median (min–max) *n* (%)	*p*-value
Demographic characteristics			
Age (years)	54.43 ± 11.51	50.93 ± 8.21	0.167a
Height (cm)	160.93 ± 6.83	160.71 ± 5.90	0.892a
Weight (kg)	73.65 ± 14.40	70.15 ± 10.59	0.272a
BMI (kg/m^2^)	28.56 ± 5.96	27.26 ± 4.60	0.336a
Marital status (married)	25 (89.3)	19 (67.9)	0.171c
Employment status (employed)	5 (15.6)	8 (25.0)	0.351c
Chronic disease (yes)	16 (50.0)	0 (0.0)	< 0.001c**
Family history of cancer (yes)	20 (62.5)	0 (0.0)	< 0.001c**
Exercise habit (yes)	9 (28.1)	5 (15.6)	0.226c
Disease-related characteristics			
Ovarian cancer subtypes	23 (71.9)	NA	NA
High-grade serous OC	2 (6.3)	NA	NA
Low-grade serous OC	3 (9.4)	NA	NA
Endometrioid OC	2 (6.3)	NA	NA
Mucinous OC	2 (6.3)	NA	NA
OC stage			
Stage 1	0 (0)	NA	NA
Stage 2	15 (46.9)	NA	NA
Stage 3	11 (34.4)	NA	NA
Stage 4	6 (18.8)	NA	NA
ECOG-PS			
0	19 (59.4)	NA	NA
1	8 (25.0)	NA	NA
2	5 (15.6)	NA	NA
4	0 (0.0)	NA	NA
5	0 (0.0)	NA	NA
Disease-free survival time (months)	24.00 (12.00–75.00)	NA	NA
Treatment			
Surgical (yes)	32 (100.0)	NA	NA
CT (yes)	32 (100.0)	NA	NA
RT (yes)	3 (9.4)	NA	NA
CT cycles	6.06 ± 1.10	NA	NA
RT cycles	0.00 (0.00–37.00)	NA	NA
Medication			
C + P	18 (56.3)	NA	NA
C + LD	14 (43.8)	NA	NA

*SD* standard deviation, min minimum, max maximum, *n* number, *BMI* body mass index, *cm* centimeter, *kg* kilogram, *m* meter, *OC* ovarian cancer, *ECOG-PS* Eastern Cooperative Oncology Group Performance Status, *CT*: chemotherapy, *RT* radiotherapy, *C* carboplatin, *P* paclitaxel, *LD* liposomal doxorubicin. ^a^Student’s *t*-test. ^b^Mann-Whitney *U*-test, *c* chi-square test, *p < 0.05, **p < 0.001, *NA* not applicable

6MWT (m) (*p* < 0.001, *r* = 1.50) and 30-CST (*p* = 0.004, *r* = 0.76) in the OC survivors were lower compared to healthy controls (Table 2). Peripheral muscle strength (*p* = 0.005, *r* = 0.72), MIP (cmH_2_O) (*p* < 0.001, *r* = 1.90), and MEP (cmH_2_O) (*p* < 0.001, *r* = 1.68) were lower in the OC survivors than those of healthy controls (Table 2). The OC survivors had lower knee extension (*p* < 0.001, *r* = 1.54) (Table 2). There was no statistically significant difference between OC survivors and healthy controls in physical activity level (*p* = 0.412, *r* < 0.01). The CIS total score in the OC survivors was significantly higher than in the healthy control group (*p* = 0.025, *r* = 0.59) (Table 2). The HADS-A score was higher in the OC survivors compared to healthy controls (*p* = 0.005, *r* = 0.75), but there was no statistically significant difference in the HADS-D score between OC survivors and healthy controls (*p* = 0.523, *r* < 0.01) (Table 2). WHO-5 total score was higher in the healthy control group than in the OC survivors (*p* = 0.008, *r* = 0.70) (Table 2). Early-stage OC survivors had significantly higher 6MWT (m) compared to advanced-stage OC survivors (*p* = 0.005, *r* = 1.83) (Table 3). Peripheral muscle strength was lower in the advanced-stage OC survivors than early-stage OC survivors (*p* = 0.013, *r* = 0.92) (Table 3). There was no significant difference in 30-CST, respiratory muscle strength, physical activity level, WHO-5 score, and CIS total score between early- and advanced-stage OC survivors (*p* > 0.05) (Table 3). FACT/GOG-NTX total scores in the early-stage OC were higher compared to advanced stage OC (*p* < 0.001, *r* = 1.42) (Table 3).

**Table 2:** Comparison of functional status and health-related parameters of ovarian cancer survivors and healthy controls

	Ovarian group (*n* = 32) Mean ± SD Median (min–max) *n* (%)	Healthy group (*n* = 32) Mean ± SD Median (min–max) *n* (%)	*p*-value	Effect size
6MWT				
6MWD (m)	465.87 ± 56.99	551.18 ± 56.82	< 0.001a**	1.50†
6MWD (% pred)	90.16 ± 13.03	100.55 ± 7.94	< 0.001a**	0.96†
30-s CST (*n*)	12.87 ± 2.75	14.71 ± 2.06	0.004a*	0.76†
Muscle strength				
Hand grip (kg)	24.25 ± 5.58	27.59 ± 3.31	0.005a*	0.72†
Knee extension (kg)	18.18 ± 4.94	24.62 ± 3.24	< 0.001a**	1.54†
Respiratory muscle strength				
MIP (cmH_2_O)	62.12 ± 18.11	92.91 ± 14.23	< 0.001a**	1.90†
MIP (% pred)	84.73 ± 23.55	123.54 ± 20.66	< 0.001a**	1.75†
MEP (cmH_2_O)	100.92 ± 35.52	151.33 ± 23.41	< 0.001a**	1.68†
MEP (% pred)	63.45 ± 18.66	92.92 ± 16.76	< 0.001a**	1.66†
Physical activity level				
IPAQ-SF (MET-min/week)	313.50 (0.00–1108.50)	270.00 (0.00–900.00)	0.412b	< 0.01§
CIS score				
Fatigue	25.50 (9.00–56.00)	15.00 (8.00–34.00)	0.016b*	0.65 †
Concentration	11.00 (5.00–35.00)	7.00 (5.00–31.00)	0.377b	0.22§
Motivation	7.50 (4.00–27.00)	6.00 (4.00–16.00)	0.074b	0.45‡
Activity	7.50 (3.00–21.00)	4.00 (3.00–13.00)	0.031b*	0.55†
Total	56.50 (23.00–118.00)	32.50 (22.00–83.00)	0.025b*	0.59†
HADS-A score HADS-D score	6.50 (0.00–18.00) 2.50 (0.00–16.00)	3.00 (0.00–9.00) 2.00 (0.00–8.00)	0.005b* 0.523b	0.75† < 0.01§
WHO-5 score	75.37 ± 18.81	86.09 ± 11.34	0.008a*	0.70†

*SD* standard deviation, min minimum, max maximum, *n* number, *IPAQ-SF* International Physical Activity Questionnaire-Short Form, *30-s CST* 30-s chair test, *6MWT* 6-min walk test, *6MWD* 6-min walk distance, *m* meter, % pred percentage of predicted value, *MIP* maximal inspiratory pressure, *MEP* maximal expiratory pressure, *cmH*_*2*_*O* centimeters of water column, *kg* kilogram, *WHO-5* World Health Organization-5, *HADS-A* Hospital Anxiety and Depression Scale-Anxiety, *HADS-D* Hospital Anxiety and Depression Scale-Depression, *CIS* Checklist Individual Strength, ^a^Student’s *t*-test, ^b^Mann-Whitney *U*-test, *p < 0.05, **p < 0.001, †large effect size (*r* = 0.50–1.0, ‡moderate effect size (*r* = 0.30–0.49), §small effect size (*r* = 0.10–0.29)

**Table 3:** Comparison of functional status and health-related parameters with early and advanced ovarian cancer survivors

	Early stage (*n* = 15) Mean ± SD Median (min–max) *n* (%)	Advanced stage (*n* = 17) Mean ± SD Median (min–max) *n* (%)	*p*-value	Effect size
Age (years)	50.80 ± 10.42	57.64 ± 11.77	0.094a	0.61†
6MWD (m)	494.90 ± 47.99	440.26 ± 52.84	0.005a*	1.83†
6MWD (% pred)	90.92 ± 14.29	89.48 ± 12.23	0.761a	0.12§
30-s CST (*n*)	13.66 ± 2.41	12.17 ± 2.92	0.129a	0.56†
Muscle strength (kg)				
Hand grip (kg)	26.79 ± 5.55	22.02 ± 4.70	0.013a*	0.92†
Knee extension (kg)	19.97 ± 4.20	16.60 ± 5.13	0.053a	0.71†
Respiratory muscle strength				
MIP (cmH_2_O)	67.20 ± 21.89	57.63 ± 13.06	0.138a	0.53†
MIP (% pred)	88.50 ± 30.23	81.40 ± 15.80	0.403a	0.30‡
MEP (cmH_2_O)	106.97 ± 31.08	95.58 ± 39.17	0.374a	0.33‡
MEP (% pred)	65.13 ± 20.66	61.96 ± 17.21	0.639a	0.17§
Physical activity level				
IPAQ-SF score (MET-min/week)	330.00 (0.00–693.00)	297.00 (0.00–1108.50)	0.970b	< 0.01§
CIS score				
Fatigue	15.00 (9.00–51.00)	28.00 (9.00–56.00)	0.030b*	0.82†
Concentration	11.00 (5.00–35.00)	11.00 (5.00–23.00)	0.970b	< 0.01§
Motivation	7.00 (4.00–27.00)	9.00 (4.00–21.00)	0.455b	0.28§
Activity	5.00 (3.00–18.00)	8.00 (3.00–21.00)	0.313b	0.38‡
Total	46.00 (25.00–118.00)	60.00 (23.00–115.00)	0.114b	0.60†
FACT/GOG-NTX				
Physical well-being	24.80 ± 2.14	21.70 ± 3.77	0.008a	1.20†
Social/family well-being	25.26 ± 1.62	24.76 ± 0.90	0.301a	0.39‡
Emotional well-being	21.73 ± 1.83	21.23 ± 2.13	0.487a	0.26§
Functional well-being	24.53 ± 2.06	21.11 ± 2.99	0.001a*	1.34†
Neurotoxicity subscale	36.60 ± 6.98	27.94 ± 6.80	0.001a*	1.26†
Total score	132.93 ± 10.89	116.76 ± 11.91	< 0.001a**	1.42†
HADS-A score	4.00 (0.00–17.00)	9.00 (1.00–18.00)	0.004b*	1.17†
HADS-D score	1.00 (0.00–16.00)	5.00 (0.00–8.00)	0.002b	1.26†
WHO-5 score	81.40 ± 16.96	70.05 ± 19.23	0.089a	0.62†

*SD* standard deviation, min minimum, max maximum, *n* number, *IPAQ-SF* International Physical Activity Questionnaire-Short Form, *30 s-CST* 30-s chair test, *6MWD* 6-min walk distance, *m* meter, % pred percentage of predicted value, *MIP* maximal inspiratory pressure, *MEP* maximal expiratory pressure, *cmH*_*2*_*O* centimeters of water column, *kg* kilogram, *WHO-5* World Health Organization-5, *HADS-A* Hospital Anxiety and Depression Scale-Anxiety, *HADS-D* Hospital Anxiety and Depression Scale-Depression, *CIS* Checklist Individual Strength, *FACT/GOG-NTX*: Neuropathy Functional Evaluation of Cancer Treatment/Gynecological Oncology-Neurotoxicity, ^a^Student’s *t*-test, ^b^Mann-Whitney *U*-test, *p < 0.05, **p < 0.001, †large effect size (*r* = 0.50–1.0), ‡moderate effect size (*r* = 0.30–0.49), §small effect size (*r* = 0.10–0.29)

## Discussion

OC is the third most common gynecologic malignancy worldwide and is associated with higher mortality rates compared to other gynecologic cancers [2]. On the other hand, advancements in surgical techniques and chemotherapy protocols have contributed to increasing survival rates and disease-free periods among OC patients in the past two decades [3]. The improved survival rates of OC patients have resulted in a need for more comprehensive assessment of treatment outcomes and an increased focus on the impact of disease-related outcomes on overall health. Our cross-sectional study revealed the following five main findings: (1) significantly lower functional exercise capacity and functional fitness and muscular endurance in OC survivors compared to healthy controls, (2) significantly lower peripheral and respiratory muscle strength, (3) significantly higher fatigue and anxiety scores, (4) similar levels of physical activity and depression, and (5) no significant difference in functional outcomes between early and advanced stage patients, except depression, anxiety, and neuropathy levels were higher in advanced stage patients. Our findings demonstrate that functional status and health-related outcomes are affected in OC survivors compared to healthy controls. Furthermore, health-related outcomes vary based on the stage of the disease.

Patients with cancer and survivors reported 6MWT distances of 403 m to 594 in previous studies [29, 30]. The 6MWT distance in this study (465.87 ± 56.99) was also in a similar range. However, the 6MWT distance from our study was significantly lower (approximately 100 m) than the 6MWT distance from a prior determinant study performed in advanced OC survivors (578.48 ± 115.33) [29]. This dissimilarity may be due to having a shorter time passed since the completion of medical treatments and a higher rate of acute side effects (fatigue, central nervous system effects, radiation pneumonia, and toxic effects) in the present study. Additionally, OC survivors reached 90% of the predicted distance in 6MWT, while the healthy age-matched control group covered 100% of the predicted distance. Previously muscle strength was demonstrated as an important predictor of 6MWT distance in OC patients [29]. The peripheral muscle strength of the OC survivors was significantly lower than the healthy controls which may have contributed to the decreased functional exercise capacity. A potential secondary factor may be weakness of the inspiratory muscles, although the relationship between MIP and exercise capacity has not been previously demonstrated in OC patients. MIP was shown as an important determinant of exercise capacity in other populations [31, 32]. Lastly, other cancer treatment-related side effects such as fatigue, dyspnea, and neuropathy are well-known phenomenon that initiates a vicious cycle leading to decreased exercise capacity in cancer patients [10, 33].

30-CST is an indicator of muscular endurance and functional fitness level in cancer survivors [19]. The mean 30-CST score obtained in our study is in line with the findings of a previous study [19] which reported a mean 30-CST score of 13.02 (3.08) s in survivors with OC. 30-CST scores of OC survivors were found to be lower compared to healthy controls in our study. Previous studies have found that 30-CST scores of elderly cancer patients were associated with functional levels, exercise capacity, and muscle strength [19, 34] which were also lower in OC survivors compared to healthy subjects in our study. Thus, given the impact of these parameters on endurance and functional fitness, the obtained 30-CST results were somewhat anticipated.

Debulking/cytoreduction surgery may lead to changes in the skeletal muscle mass and body fat volume [35]. A previous study has reported that compared to healthy control, OC patients/survivors have 29 to 50% lower muscle mass [35]. Our study also revealed a significant difference in knee extension strength in OC patients compared to healthy controls. A recent study demonstrated a significant decrease in hand grip muscle strength among cancer patients compared to healthy individuals (25 ± 9.0 kg and 29 ± 8.0 kg, respectively) [36]. Our study similarly revealed a significant disparity in hand grip strength between healthy controls and OC survivors. However, a prior study examining patients with advanced OC survivors and healthy controls reported comparable hand grip and knee extension strength values (cancer patients: 24.4 ± 6.6 kg; 24.1 ± 9.1 kg, healthy controls: 26.8 ± 7.0; 25.2 ± 8.4 kg respectively) [16]. The high SD values indicating a heterogeneous study population and small sample size (cancer group *n* = 20; control group *n* = 20) may explain no significant difference in the hand grip strength comparison between the groups in this study. On the other hand, the hand grip strength value we obtained from patients with advanced OC survivors (22.02 ± 4.70 kg) was relatively consistent with the findings of this study [16]. A previous study assessed the hand grip strength of advanced OC survivors (24.13 ± 6.0), and similar results were obtained in our study in advanced-stage OC survivors [29]. Furthermore, this present study revealed advanced-stage OC survivors had a significant reduction in hand grip strength compared to early-stage OC survivors. One potential explanation for this finding may be the loss of muscle mass due to cancer-related factors which is common in patients with advanced-stage cancer patients [8, 37]. Additionally, another potential explanation for this finding is a doxorubicin (Adriamycin) chemotherapeutic drug, commonly used in advanced OC patients and was found to cause permanent muscle necrosis, changes in myofilament structure, and reduced muscle strength in a murine model study [38].

OC patients may have tumors involving the diaphragm, intestine mesentery, and portal triad [2]. Approximately half of OC patients had tumor operations involving the upper abdomen quadrant up to the omentum majus [39]. It has been reported that the incidence of respiratory muscle dysfunction after upper abdominal surgery can range between 20 and 40% [39]. The resection of the diaphragm as part of cytoreductive surgery may also be a contributing factor to respiratory muscle dysfunction. Moreover, chemotherapy commonly leads to dyspnea and exercise intolerance, which may be indicating a potential effect on the impairment of inspiratory muscle contractile function [8]. Altogether, the combination of these factors may have a potential detrimental impact on the respiratory muscle strength of OC patients/survivors. However, the alterations in respiratory muscle function are yet to be determined. This present study showed decreased respiratory muscle strength in OC survivors compared to healthy controls. Additionally, the fact that survivors achieved only 84% of the predicted MIP value might have contributed to the observed reduction in exercise capacity. The MEP was also 63% of the predicted value, possibly due to abdominal muscle weakness induced by the surgery. Although the subgroup analysis in the present study revealed no significant difference between the groups in respiratory muscle strength, a mean difference of approximately 10 cmH_2_O was observed in respiratory muscle strength (MIP and MEP) values between early- and advanced-stage OC survivors. The moderate effect size of the difference between the groups in this study suggests the absence of significant differences in the secondary analysis may be attributed to the relatively small sample size. Therefore, studies with larger sample size studies may be helpful for enhancing the body of evidence on this subject.

The American Cancer Society (ACS) and the American College of Sports Medicine (ACSM) recommend 150–300 min of moderate or 75–150 min of vigorous exercise per week for cancer survivors to improve health-related parameters (quality of life, functional mobility, fatigue level, exercise capacity, etc.) [12, 40]. On the other hand, it was recently reported that most OC survivors had lower physical activity levels than recommended [16, 41]. Similarly, the physical activity level of OC survivors was below the recommended level in our study. A previous study assessing the physical activity levels of advanced and early-stage OC patients found no difference between the groups [41]. Likewise, no difference was observed in the physical activity levels of the patients in subgroup analysis according to the stage in our study.

Fatigue is a prevalent and persistent symptom with a high prevalence rate of 93% reported by patients with OC [33]. This debilitating condition often continues to affect patients even after completing their treatment [33]. Compared to controls, OC survivors present persistent long-term fatigue [42]. Furthermore, an association between neurotoxicity and long-term fatigue in OC survivors was demonstrated [33]. OC survivors had severe fatigue compared to healthy controls in the present study. Our results also demonstrated that advanced-stage OC survivors had experienced higher levels of fatigue similar to the previous findings on this subject [42]. Muscle strength, neuropathy, mobility, depression, anxiety, and exercise intolerance may all contribute to fatigue [7, 33], which may explain the difference between OC survivor subgroups.

The chemotherapy agents most commonly used in the treatment of OC patients are platinum compounds (usually cisplatin or carboplatin), especially cisplatin is known to cause neurotoxicity, inducing mainly sensory neuropathy of the upper and lower extremities [2]. Neuropathy approximately impacts more than 40% of OC patients and may still persist following the treatment process in cancer survivors [43]. A previous study in OC patients/survivors assessed the neuropathy experienced by the patients during their treatment with chemotherapy agents and during follow-up [43]. The study demonstrated that majority of patients experienced neuropathy symptoms during the 9-month follow-up period; this condition affected the functional and physical ability of the patient [43]. An important portion of OC survivors in the present study also suffered from neuropathy even after 12 months after the completion of adjuvant treatments. Additionally, patients with advanced-stage OC survivors were observed to be more affected by neuropathy symptoms than patients with early-stage OC survivors in the present study. The cumulative effect of repeated exposure to chemotherapy drugs leading to more severe neuropathy symptoms in advanced-stage patients may have played a role in this result [44]. Higher doses of chemotherapy can cause greater nerve damage, resulting in more severe and persistent neuropathic symptoms [44]. The minimal clinically important difference (MCID) of the FACT/GOG-NTX in a previous study of patients with cancer was calculated as 3.68 [44]. The difference between mean scores regarding neuropathy symptoms was almost two times higher than the MCID value between advanced and early-stage patients in the present study which suggests a clinically significant difference in neuropathy in early- and advanced-stage OC.

OC patients experience depression, anxiety, and reduced quality of life due to fear of recurrence, persistent fatigue, sexual inactivity, and repetitive treatment cycles [15, 45]. The incidence of anxiety disorders among OC patients/survivors is approximately 3.5 times higher in the first 2 years following diagnosis [45]. Although the anxiety levels of the OC survivors in our study were significantly higher than the healthy controls, HADS-A scores were lower than the previously reported cut-off scores (a cut-off score > 9 is used to identify higher anxiety for cancer patients) [46]. The potential explanation may be that the anxiety levels of advanced cancer patients were above the cut-off value, but the anxiety levels of early-stage cancer patients were below the cut-off value. Furthermore, a significant statistical difference in depression and anxiety levels was found between early-stage and advanced-stage OC patients. Advanced-stage OC patients exhibited higher anxiety levels, likely due to increased fear of cancer recurrence and the burden of recurrent disease cycles [45]. Additionally, anxiety levels in advanced-stage OC patients were above the cut-off value for cancer patients. Prior research demonstrated no difference in the quality of life of early- and advanced-stage OC patients [47]. We similarly observed no significant difference in the generic quality of life between the early- and advanced-stage OC patients of our study. On the other hand, our study showed a significant diminish in generic quality of life compared to OC survivors and age-matched healthy controls.

The strength of this study is the evaluation of OC survivors with a comprehensive approach regarding health-related and functional parameters and the employment of objective methods for assessing functionality and the majority of health-related parameters. The present study also has some limitations. Firstly, the relatively small sample size may have reduced the representativeness of the results, particularly in the subgroup analysis. Secondly, due to the cross-sectional design, the variability of disease-related symptoms could not be observed. A future longitudinal study after completing the treatment cycles may be helpful for monitoring the changes in functional status and health-related parameters.

## Conclusion

OC survivors reported lower functional exercise capacity, muscle endurance and functional fitness, respiratory muscle strength, peripheral and lower extremity muscle strength, quality of life, and higher levels of fatigue and anxiety than healthy controls in this study. Advanced-stage OC survivors had higher levels of fatigue, neuropathy, anxiety, depression, decreased exercise capacity, and peripheral muscle strength than early-stage OC survivors. The present study findings may be important to fill the gaps regarding the functional status and health-related parameters characteristics of OC survivors and may contribute to the development of tailored cancer rehabilitation program.

## Data Availability

The study data is available upon reasonable request from the corresponding author.

## References

[1] Sung H, Ferlay J, Siegel RL, Laversanne M, Soerjomataram I, Jemal A, Bray F (2021). Global cancer statistics 2020: GLOBOCAN estimates of incidence and mortality worldwide for 36 cancers in 185 countries. CA Cancer J Clin.

[2] Armstrong DK, Alvarez RD, Bakkum-Gamez JN, Barroilhet L, Behbakht K, Berchuck A, Chen LM, Cristea M, DeRosa M, Eisenhauer EL (2021). Ovarian cancer, version 2.2020, NCCN Clinical Practice Guidelines in Oncology. J Natl Compr Canc Netw.

[3] Lheureux S, Gourley C, Vergote I, Oza AM (2019). Epithelial ovarian cancer. Lancet.

[4] Campbell R, King MT, Stockler MR, Lee YC, Roncolato FT, Friedlander ML (2023). Patient-reported outcomes in ovarian cancer: facilitating and enhancing the reporting of symptoms, adverse events, and subjective benefit of treatment in clinical trials and clinical practice. Patient Relat Outcome Meas.

[5] Cuzick J (2023). The importance of long-term follow up of participants in clinical trials. Br J Cancer.

[6] Meraner V, Gamper EM, Grahmann A, Giesinger JM, Wiesbauer P, Sztankay M, Zeimet AG, Sperner-Unterweger B, Holzner B (2012). Monitoring physical and psychosocial symptom trajectories in ovarian cancer patients receiving chemotherapy. BMC Cancer.

[7] Gernier F, Ahmed-Lecheheb D, Pautier P, Floquet A, Nadeau C, Frank S, Alexandre J, Selle F, Berton-Rigaud D, Kalbacher E (2021). BMC Cancer.

[8] Gilliam LA, St Clair DK (2011). Chemotherapy-induced weakness and fatigue in skeletal muscle: the role of oxidative stress. Antioxid Redox Sig.

[9] Vitarello J, Goncalves MD, Zhou QC, Iasonos A, Halpenny DF, Plodkowski A, Schwitzer E, Mueller JJ, Zivanovic O, Jones LW (2021). The effects of neoadjuvant chemotherapy and interval debulking surgery on body composition in patients with ovarian cancer. JCSM Clin Rep.

[10] Parashar S, Akhter N, Paplomata E, Elgendy IY, Upadhyaya D, Scherrer-Crosbie M, Okwuosa TM, Sanghani RM, Chalas E, Lindley KJ (2023). Cancer treatment-related cardiovascular toxicity in gynecologic malignancies: JACC: cardiooncology state-of-the-art review. JACC CardioOncol.

[11] Zinger A, Cho WC, Ben-Yehuda A (2017) “Cancer and aging - the inflammatory connection.” Aging Dis 8 611–27. 10.14336/AD.2016.1230https://www.ncbi.nlm.nih.gov/pubmed/28966805 10.14336/AD.2016.1230 PMC5614325 28966805

[12] Rock CL, Thomson C, Gansler T, Gapstur SM, McCullough ML, Patel AV, Andrews KS, Bandera EV, Spees CK, Robien K (2020). American Cancer Society guideline for diet and physical activity for cancer prevention. CA Cancer J Clin.

[13] Schofield C, Newton RU, Galvao DA, Cohen PA, Peddle-McIntyre CJ (2017). A physiological profile of ovarian cancer survivors to inform tailored exercise interventions and the development of exercise oncology guidelines. Int J Gynecol Cancer.

[14] Naughton MJ, Weaver KE (2014) “Physical and mental health among cancer survivors: considerations for long-term care and quality of life.” N C Med J 75 283–6. 10.18043/ncm.75.4.283. (https://www.ncbi.nlm.nih.gov/pubmed/25046097) 10.18043/ncm.75.4.283 PMC4503227 25046097

[15] Stein KD, Syrjala KL, Andrykowski MA (2008). Physical and psychological long-term and late effects of cancer. Cancer.

[16] Schofield C, Newton RU, Cohen PA, Galvao DA, McVeigh JA, Hart NH, Mohan GR, Tan J, Salfinger SG, Straker LM (2018). Activity behaviors and physiological characteristics of women with advanced-stage ovarian cancer: a preliminary cross-sectional investigation. Int J Gynecol Cancer.

[17] Breithaupt-Groegler K, Coch C, Coenen M, Donath F, Erb-Zohar K, Francke K, Goehler K, Iovino M, Kammerer KP, Mikus G (2017). Who is a ‘healthy subject’?-consensus results on pivotal eligibility criteria for clinical trials. Eur J Clin Pharmacol.

[18] ATS Committee on Proficiency Standards for Clinical Pulmonary Function Laboratories (2002) ATS statement: guidelines for the six-minute walk test. Am J Respir Crit Care Med 166(1):111–117. 10.1164/ajrccm.166.1.at1102 10.1164/ajrccm.166.1.at1102 12091180

[19] Blackwood J, Rybicki K (2021) “Physical function measurement in older long-term cancer survivors.” J Frailty Sarcopenia Falls 6 139–46. 10.22540/JFSF-06-139. (https://www.ncbi.nlm.nih.gov/pubmed/34557613) 10.22540/JFSF-06-139 PMC8419850 34557613

[20] Sousa-Santos AR, Amaral TF (2017). Differences in handgrip strength protocols to identify sarcopenia and frailty - a systematic review. BMC Geriatr.

[21] Mentiplay BF, Perraton LG, Bower KJ, Adair B, Pua YH, Williams GP, McGaw R, Clark RA (2015). Assessment of lower limb muscle strength and power using hand-held and fixed dynamometry: a reliability and validity study. PLoS One.

[22] Saglam M, Arikan H, Savci S, Inal-Ince D, Bosnak-Guclu M, Karabulut E, Tokgozoglu L (2010). International physical activity questionnaire: reliability and validity of the Turkish version. Percept Mot Skills.

[23] Api M, Purut YE, Akiş S, Keleş E, Ceylan Y, Kocakuşak CK, Giray B, Uzun MG (2019) Validation of Turkish version of Eastern Cooperative Oncology Group Performance Status (ECOG-PS) for gynecologic oncology patients. Int J Gynecol Cancer 29:A617. 10.1136/ijgc-2019-ESGO.1236

[24] Ergin G, Yildirim Y (2012). A validity and reliability study of the Turkish Checklist Individual Strength (CIS) questionnaire in musculoskeletal physical therapy patients. Physiother Theory Pract.

[25] Calhoun EA, Welshman EE, Chang CH, Lurain JR, Fishman DA, Hunt TL, Cella D (2003). Psychometric evaluation of the Functional Assessment of Cancer Therapy/Gynecologic Oncology Group-Neurotoxicity (FACT/GOG-NTx) questionnaire for patients receiving systemic chemotherapy. Int J Gynecol Cancer.

[26] Rodgers J, Martin CR, Morse RC, Kendell K, Verrill M (2005). An investigation into the psychometric properties of the Hospital Anxiety and Depression Scale in patients with breast cancer. Health Qual Life Outcomes.

[27] Eser E, Cevik C, Baydur H, Gunes S, Esgin TA, Oztekin CS, Eker E, Gumussoy U, Eser GB, Ozyurt B (2019). Reliability and validity of the Turkish version of the WHO-5, in adults and older adults for its use in primary care settings. Prim Health Care Res Dev.

[28] Leppink J, O’Sullivan P, Winston K (2016). Effect size - large, medium, and small. Perspect Med Educ.

[29] Kizilirmak AS, Karadibak D, Gultekin SC, Ozsoy I, Yavuzsen HT, Yavuzsen T, Oztop I (2023). Predictors of the 6-min walk test in patients with ovarian cancer. Support Care Cancer.

[30] Schmidt K, Vogt L, Thiel C, Jager E, Banzer W (2013). Validity of the six-minute walk test in cancer patients. Int J Sports Med.

[31] van der Esch M, van’t Hul AJ, Heijmans M, Dekker J (2004) “Respiratory muscle performance as a possible determinant of exercise capacity in patients with ankylosing spondylitis.” Aust J Physiother 50 41-5. 10.1016/s0004-9514(14)60247-6. (https://www.ncbi.nlm.nih.gov/pubmed/14987191) 10.1016/s0004-9514(14)60247-6 14987191

[32] Ferrer-Sargues FJ, Peiro-Molina E, Salvador-Coloma P, Carrasco Moreno JI, Cano-Sanchez A, Vazquez-Arce MI, Insa Albert B, Sepulveda Sanchis P, Cebria IIMA (2020) Cardiopulmonary rehabilitation improves respiratory muscle function and functional capacity in children with congenital heart disease. A prospective cohort study. Int J Environ Res Public Health 17. 10.3390/ijerph17124328 (https://www.ncbi.nlm.nih.gov/pubmed/32560441) 10.3390/ijerph17124328 PMC7345179 32560441

[33] Thong MSY, van Noorden CJF, Steindorf K, Arndt V (2020). Cancer-related fatigue: causes and current treatment options. Curr Treat Options Oncol.

[34] Harrington S, Lee J, Colon G, Alappattu M (2016). Oncology section edge task force on prostate cancer: a systematic review of outcome measures for health-related quality of life. Rehabil Oncol.

[35] Nakayama N, Nakayama K, Ishibashi T, Katayama S, Kyo S (2022) Effect of muscle loss but not fat loss during primary debulking surgery and chemotherapy on prognosis of patients with ovarian cancer. J Clin Med 11. 10.3390/jcm11113184 (https://www.ncbi.nlm.nih.gov/pubmed/35683568) 10.3390/jcm11113184 PMC9181028 35683568

[36] Duarte ACF, Silva BA, Avelino PR, de Menezes KKP (2021). “Grip strength, functional capacity, and quality of life of individuals with cancer”. Fisioterapia e Pesq.

[37] Kilgour RD, Vigano A, Trutschnigg B, Lucar E, Borod M, Morais JA (2013) Handgrip strength predicts survival and is associated with markers of clinical and functional outcomes in advanced cancer patients. Support Care Cancer 21:3261–70. 10.1007/s00520-013-1894-4 (https://www.ncbi.nlm.nih.gov/pubmed/23872952) 10.1007/s00520-013-1894-4 23872952

[38] Gilliam LA, Moylan JS, Callahan LA, Sumandea MP, Reid MB (2011). Doxorubicin causes diaphragm weakness in murine models of cancer chemotherapy. Muscle Nerve.

[39] Zivanovic O, Sima CS, Iasonos A, Hoskins WJ, Pingle PR, Leitao MM, Sonoda Y, Abu-Rustum NR, Barakat RR, Chi DS (2010). The effect of primary cytoreduction on outcomes of patients with FIGO stage IIIc ovarian cancer stratified by the initial tumor burden in the upper abdomen cephalad to the greater omentum. Gynecol Oncol.

[40] Campbell KL, Winters-Stone KM, Wiskemann J, May AM, Schwartz AL, Courneya KS, Zucker DS, Matthews CE, Ligibel JA, Gerber LH (2019). Exercise guidelines for cancer survivors: consensus statement from international multidisciplinary roundtable. Med Sci Sports Exerc.

[41] Jones T, Sandler C, Vagenas D, Janda M, Obermair A, Hayes S (2021). Physical activity levels among ovarian cancer survivors: a prospective longitudinal cohort study. Int J Gynecol Cancer.

[42] Wang XS, Woodruff JF (2015). Cancer-related and treatment-related fatigue. Gynecol Oncol.

[43] Pignata S, De Placido S, Biamonte R, Scambia G, Di Vagno G, Colucci G, Febbraro A, Marinaccio M, Lombardi AV, Manzione L (2006). Residual neurotoxicity in ovarian cancer patients in clinical remission after first-line chemotherapy with carboplatin and paclitaxel: the Multicenter Italian Trial in ovarian cancer (MITO-4) retrospective study. BMC Cancer.

[44] Cheng HL, Lopez V, Lam SC, Leung AKT, Li YC, Wong KH, Au JSK, Sundar R, Chan A, De Ng TR (2020). Psychometric testing of the Functional Assessment of Cancer Therapy/Gynecologic Oncology Group-Neurotoxicity (FACT/GOG-NTX) subscale in a longitudinal study of cancer patients treated with chemotherapy. Health Qual Life Outcomes.

[45] Hu S, Baraghoshi D, Chang CP, Rowe K, Snyder J, Deshmukh V, Newman M, Fraser A, Smith K, Peoples AR (2023). Mental health disorders among ovarian cancer survivors in a population-based cohort. Cancer Med.

[46] Annunziata MA, Muzzatti B, Bidoli E, Flaiban C, Bomben F, Piccinin M, Gipponi KM, Mariutti G, Busato S, Mella S (2020). Hospital Anxiety and Depression Scale (HADS) accuracy in cancer patients. Support Care Cancer.

[47] Mirabeau-Beale KL, Kornblith AB, Penson RT, Lee H, Goodman A, Campos SM, Duska L, Pereira L, Bryan J, Matulonis UA (2009). Comparison of the quality of life of early and advanced stage ovarian cancer survivors. Gynecol Oncol.

